# Normalization of GC-MS Metabolomics Data in Adherent Cells: A Practical Comparison of Approaches

**DOI:** 10.3390/ijms27073219

**Published:** 2026-04-02

**Authors:** Ilya Yu. Kurbatov, Svyatoslav V. Zakharov, Olga I. Kiseleva, Viktoriia A. Arzumanian, Igor V. Vakhrushev, Roza Yu. Saryglar, Victoria D. Novikova, Yan S. Kim, Ekaterina V. Poverennaya

**Affiliations:** Institute of Biomedical Chemistry, Pogodinskaya Street, 10, 119121 Moscow, Russia; kurbatild@gmail.com (I.Y.K.); zssvyat35@gmail.com (S.V.Z.); arzumanian.victoria@gmail.com (V.A.A.); vakhrunya@gmail.com (I.V.V.); roza_saryglar@mail.ru (R.Y.S.); vnlvikova@gmail.com (V.D.N.); yankimhcc@gmail.com (Y.S.K.);

**Keywords:** adherent cell lines, GC × GC-MS, metabolomics, DNA concentration normalization, total protein normalization, extracted ion current normalization

## Abstract

Data compatibility remains a major challenge in metabolomics, as commonly used measures of biological material—such as sample weight or cell count—are often poorly reproducible. Here, we systematically evaluated practical normalization strategies for GC × GC-MS-based metabolomic profiling of two widely used model cell lines: human hepatoblastoma (HepG2) and mesenchymal stromal cells (MSCs). We compared orthogonal biomass estimates, including total protein and double-stranded DNA quantified either directly in aliquots of the cell suspension lysate aliquots or in the post-extraction cell precipitate, alongside normalization based on extracted ion current (XIC). We also assessed three widely used extraction mixtures—methanol/chloroform/water (7:2:1); methanol/water (8:2); acetonitrile/isopropanol/water (3:3:2)—for metabolome coverage and normalization robustness. Under realistic biological variability, signal-to-biomass dependencies were moderate. In contrast, under strictly controlled conditions, DNA- and protein-based normalization yielded near-linear relationships with metabolite abundances (R^2^ > 0.90), demonstrating that biological variability is the dominant source of dispersion rather than technical factors. Methanol/chloroform/water system provided the broadest metabolome coverage and strongest correlation with injected biomass. Based on these findings, we recommend normalization to total precipitate protein or DNA using the methanol/chloroform/water extraction protocol, with XIC as a complementary quality control metric.

## 1. Introduction

Normalization is a critical step in omics research, ensuring data comparability across samples and downstream analyses. In metabolomic profiling of biofluids, normalization is relatively straightforward, as the volume of the analyzed aliquot is precisely defined. In the case of urine, normalization to endogenous creatinine is well established. For cell lines, tissues, or small organisms, however, no universally accepted strategy exists. Current approaches typically normalize metabolite levels to sample mass [1] or cell number [2,3]. Nevertheless, the reliability of these methods in the context of true biological variability remains uncertain.

Normalization by cell count [3], despite its intuitive appeal, is associated with significant methodological limitations. Cell counting is inherently susceptible to between-sample variability, arising from uneven cell distribution in suspension and the imprecision of manual counting. Furthermore, adhesive cell lines are prone to forming cell clusters, which can further compromise counting accuracy and reproducibility [4,5]. The presence of such cell aggregates can distort estimates of the true amount of biological material presented in the sample. These limitations have motivated the development of alternative normalization strategies that relate to cellular biomass more robustly than cell number alone. In metabolomics, a range of normalization approaches based on biochemical and analytical parameters are currently applied [3,6,7]. The most widely used ones include normalization by ion current [8], as well as by total protein and DNA content [9,10,11,12].

Normalization to the summed extracted ion current (XIC) offers a distinct practical advantage over the approaches described above: it requires no additional sample manipulation and can be applied retrospectively at the data-processing stage, making it particularly valuable for the re-analysis of previously acquired or publicly available datasets. However, the universal application of this method is constrained by an important limitation. According to available data, XIC-based normalization is only reliable when the amount of biological material across samples differs by no more than twofold [3].

Normalization to total protein or DNA content is widely recognized as the most promising and versatile approach. Studies have demonstrated that, when using both approaches, deviations from the experimental mean value amounted to less than 20% for samples containing more than 0.5 million cells [9]. In addition, the use of protein normalization can achieve a coefficient of variation below 30% for more than 300 metabolites [2].

Despite the demonstrated effectiveness, these methods remain insufficiently standardized. No universally accepted normalization standard has been established in metabolomics. Research groups currently either follow in-house protocols or omit normalization entirely. A systematic comparison of the accuracy, efficiency, and comparability of the available normalization approaches is also lacking. This absence of consensus hampers robust interlaboratory comparison of metabolomics data and limits the reproducibility of findings across studies.

In this work, we attempted to select the optimal normalization strategy from among the most commonly used approaches, in order to improve the quality of metabolomic profiling data acquired by GC × GC-MS. To this end, we systematically evaluated the reproducibility of several biomass estimation methods in the context of three commonly used extraction mixtures, and assessed their performance under conditions of biological heterogeneity introduced by culturing HepG2 human hepatoblastoma cells in parallel on independent culture plates.

## 2. Results and Discussion

### 2.1. Optimization of Extraction Solvent and Evaluation of Protein- and DNA-Based Normalization for GC × GC-MS Metabolomic Profiling of HepG2 Cells

The first stage of the study addressed a key methodological question: whether metabolomic data can be reliably normalized to protein or DNA content under extraction conditions compatible with gas chromatography. Specifically, we assessed whether quantification of protein and DNA content could be accurately quantified in the solid residue remaining after metabolite extraction. Implementation of such an approach would enable simultaneous acquisition of biomass estimates and metabolomic profiles within a single experiment, eliminating the need for additional sample processing steps.

To this end, the HepG2 cells were cultured under standardized conditions, with the sole variable being the serum source, supplied by three different manufacturers. Within the same experimental framework, three solvent systems widely used in GC-MS-based metabolomics were evaluated: methanol/chloroform/water (7:2:1, *v*:*v*:*v*) [3], methanol/water (8:2, *v*:*v*:*v*) [13], and acetonitrile/isopropanol/water (3:3:2, *v*:*v*:*v*) [14]. Solvent composition was considered a critical variable, as it directly influences both the efficiency and selectivity of metabolite extraction and the co-precipitation of proteins and DNA. The primary objective of this stage was to identify the extraction mixture most compatible with protein- and DNA-based normalization from cell precipitates. Metabolites were extracted from samples cultured under three serum conditions (n = 9 per solvent), yielding 27 samples in total.

**Figure 1:**
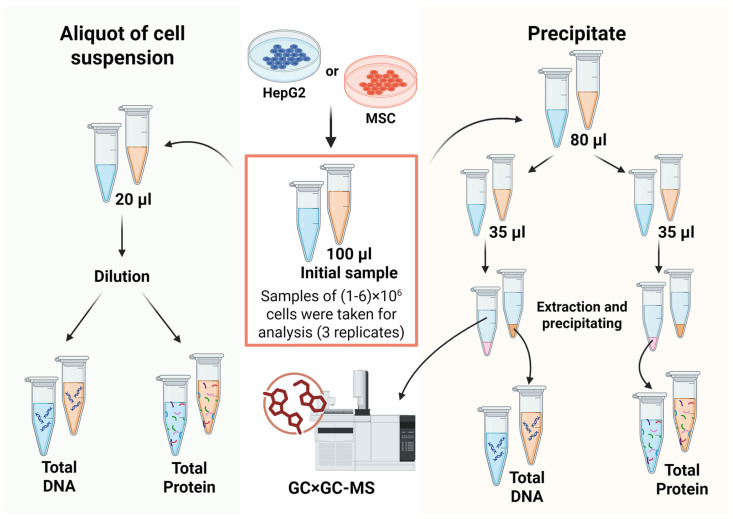
Schematic overview of the analytical workflow. A 20 µL aliquot of cell suspension was withdrawn from the initial 100 µL cell suspension aliquot for direct protein and DNA quantification. The remaining 80 µL of the cell suspension was split into two 35 µL aliquots. Metabolites were extracted from each aliquot of cell suspension using one of three extraction solvents. The resulting precipitates were dried, and total protein was quantified in one replicate, while DNA content was determined from the other.

As described in Section 2.3 and Section 2.4, each initial sample was divided into three aliquots of cell suspension: one 20 μL aliquot for direct protein and DNA quantification, and two 35 μL aliquots for metabolite extraction and recovery of the cellular precipitate (Figure 1). This experimental design yielded two independent estimates of biological polymer content from the same sample, alongside two parallel metabolomic profiles—enabling internal consistency checks and improving the robustness of the normalization assessment.

Comparison of the three extraction systems revealed a pronounced effect on metabolite coverage. Among the tested mixtures, the methanol/chloroform/water system yielded the highest number of consistently detected metabolites (97), outperforming methanol/water (90) and acetonitrile/isopropanol/water (88), making it the preferred choice when broad metabolomic coverage is the primary objective.

Among the three extraction systems, methanol/chloroform/water also yielded the most favorable normalization performance. This mixture produced the highest mean coefficients of determination (R^2^) between metabolite signal intensities and biological material content—whether quantified as protein or DNA—along with the greatest proportion of metabolites with R^2^ > 0.75 (Table 1).

The methanol/water solvent system also yielded high coefficients of determination, indicating good extraction reproducibility across a substantial fraction of metabolites; however, overall metabolome coverage was moderately reduced under these conditions. Methanol/chloroform/water mixture was therefore selected as the primary extraction solvent for all subsequent experiments, as it provided both maximal metabolite coverage and the strongest correlation between metabolite signal intensity and protein/DNA concentration in the post-extraction precipitate.

Correlation analysis between metabolite peak areas and different normalization factors—including protein or DNA concentrations determined under various conditions, as well as the summed XIC of detected metabolites—revealed substantial differences in the performance of the applied normalization strategies. Compared with protein-based normalization, DNA-based normalization of direct lysate aliquots was associated with substantially lower mean R^2^ values and a reduced proportion of metabolites with R^2^ > 0.75 (Table 1). We attribute this primarily to the low DNA amounts present in lysate aliquots (on the order of ng/μL), which increases susceptibility to pipetting errors and nonspecific adsorption. Additionally, DNA determinations were performed in standard polypropylene tubes rather than low DNA-binding consumables, which may have further contributed to nonspecific DNA loss. Together, these factors likely amplified technical variability in lysate-based DNA measurements, while both DNA- and protein-based metrics from the post-extraction cell pellet remained markedly more robust.

**Figure 2:**
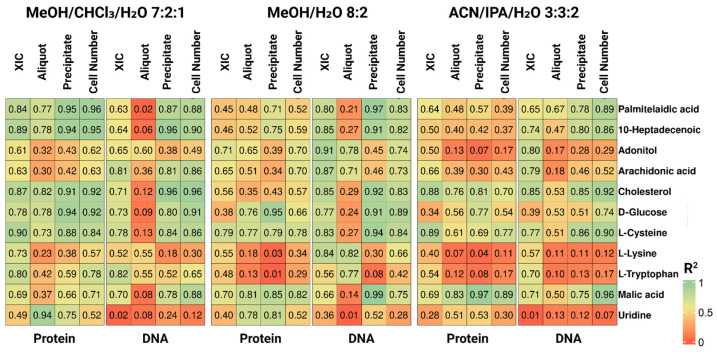
Coefficients of determination (R^2^) between peak areas of 11 selected metabolites and six normalization parameters—protein and DNA concentrations in precipitates and aliquots of cell suspension, XIC, and nominal number of cells from protein-extract and DNA-extract—across three extraction solvents.

The data suggest that the effectiveness of a given normalization factor depends on the chemical nature of the metabolite. To enable consistent comparisons across this and subsequent experiments, eleven metabolites representing distinct chemical classes were selected for detailed analysis. Selection was guided by two complementary considerations: (i) GC × GC-MS method characteristics—specifically, thermostability and compatibility with silylation derivatization, which targets polar functional groups to render otherwise non-volatile compounds amenable to analysis—and (ii) the typical metabolome composition of adherent cell lines, with particular reference to HepG2 cells [15]. All selected metabolites met the detection and quantification thresholds defined in the Methods, and were verified to be consistently detected across all cell loading levels within each experiment. Together, they represent the major chemical classes detectable under our GC × GC-MS conditions, including fatty acids and conjugates, carbohydrates, amino acids and related derivatives, beta-hydroxy acids, cholestane steroids, and pyrimidine nucleosides. Figure 2 presents the coefficients of determination between each normalization factor and the metabolite signal for these representative compounds across the full dynamic range of biomass loads tested. Among the evaluated parameters, protein content measured in cell precipitates after metabolite extraction yielded the strongest and most reproducible correlations (mean R^2^ = 0.66 ± 0.24, mean ± SD) in the methanol/chloroform/water system. In contrast, DNA quantified from the precipitate showed the greatest variability (mean R^2^ = 0.64 ± 0.30) in two solvents, indicating lower reliability as a normalization factor for the metabolite set under study.

Analysis of individual compounds revealed a clear pattern: metabolites exhibiting strong correlations with precipitate protein content generally showed high correlations with precipitate DNA. For example, cysteine displayed an R^2^ of 0.88 and 0.84 with precipitate protein and precipitate DNA, respectively, whereas its correlation with DNA quantified from the cell suspension aliquot was markedly lower (R^2^ = 0.13). This disparity suggests that the post-extraction precipitate may better preserve the quantitative relationship between metabolites and cellular biomass than the lysate.

Notably, correlations between metabolite peak areas and the evaluated normalization factors remained moderate even for the most favorable extraction solvent system (0.64–0.66 on average for precipitate-based normalization). Several complementary factors likely contribute to this. First, the efficiency and reproducibility of DNA and protein precipitation may vary across extraction solvents depending on their physicochemical properties, introducing heterogeneity in the amount of material recovered for quantification even when the initial cell number is held constant. Second, manual aliquoting—performed both for protein/DNA determination and metabolite extraction—may introduce variability in volume and sample representativeness, particularly given the potential inhomogeneity or partial sedimentation of the cell suspension between preparation and aliquoting. Third, despite the use of a standardized cell line, metabolic activity may vary during culture, introducing intra-experimental heterogeneity whose magnitude remains to be systematically assessed.

These observations highlight that while the methanol/chloroform/water (7:2:1) system offers optimal compatibility with pellet-based normalization under realistic culture conditions, the moderate R^2^ values suggest contributions from both technical factors—such as precipitation efficiency and aliquoting variability—and uncontrolled biological heterogeneity. To decouple these effects, we systematically evaluated normalization performance across a series of progressively more complex experimental settings: first, by assessing the reproducibility of biopolymer precipitation across solvents in the complete absence of biological variation (Section 2.2); then, by metabolomic profiling under strictly controlled cell loading (Section 2.3); and finally, under conditions of minimal but controlled biological variability (Section 2.4). This stepwise experimental design allows the relative contributions of technical and biological factors to normalization performance to be clearly resolved.

### 2.2. Technical Reproducibility of Protein and DNA Precipitation Across Extraction Solvents

Although the first experiment established a relationship between biomass and metabolite peak areas, it did not clarify the underlying causes of the limited correlations observed. To determine the extent to which this variability could be attributed to technical aspects of sample preparation—specifically, the completeness of DNA and protein precipitation and the homogeneity of aliquoting—a dedicated follow-up experiment was conducted.

To directly evaluate the variability in protein and DNA precipitation independently of fluctuations in the initial biomass, a series of parallel determinations was conducted. From a single homogeneous cell suspension (approximately 21 million cells), 18 equal-volume aliquots were collected and distributed evenly among the three extraction solvent systems (n = 6 samples per solvent). Each resulting precipitate was subjected to parallel quantification of protein (BCA assay, n = 3) and DNA (PicoGreen assay, n = 3), enabling simultaneous assessment of both the stability of the precipitation process itself and the reproducibility of the analytical measurements.

The results of protein and DNA quantification across the three solvent systems are summarized in Table 2.

These results indicate that sample preparation reproducibility was substantially higher than initially anticipated. The methanol/chloroform/water (7:2:1) extraction system exhibited an optimal balance between maximal protein recovery (956.54 μg/mL) and low technical variability (protein CV: 0.99%; DNA CV: 4.35%), supporting both broad metabolite coverage and workflow robustness. The high reproducibility observed under these standardized conditions suggests that the moderate signal-to-biomass correlations in the first experiment are largely attributable not to technical limitations of sample preparation but to intrinsic biological heterogeneity of the samples, likely arising from variations in reagents and culture conditions.

### 2.3. Technical Performance of Normalization Metrics in Pooled MSCs in the Absence of Biological Variation

Building on the findings of the first experiment, a follow-up metabolomic study was conducted to assess whether the normalization factors identified are generalizable across cell types. Mesenchymal stromal cells (MSCs) were selected as an alternative adherent cell model, allowing us to verify that the performance of DNA- and protein-based normalization is not restricted to HepG2 cells but reflects a broader applicability. Unlike the initial HepG2 experiment, which was potentially confounded by variability in culture conditions between individual dishes, the present experiment employed a strict unification strategy. MSCs were cultured under fully identical conditions, including the same culture medium and reagent suppliers, identical seeding density, and synchronized cultivation timelines.

Upon reaching the target cell number (ca. 35 million cells), all biological material was pooled into a single suspension, eliminating inter-dish variability in metabolic state, differentiation status, and other cellular characteristics. Cycloserine was added to the pool as an internal standard to monitor the homogeneity and reproducibility of the subsequent aliquoting process. Equal-volume aliquots containing ca. 2, 3, and 6 million cells in 100 μL of phosphate-buffered saline were manually collected. Subsequent sample preparation followed the same workflow as in the first experiment, with the sole exception that only a single extraction solvent—methanol/chloroform/water (7:2:1), previously identified as optimal—was used.

First, we assessed the concordance between measured quantitative parameters and nominal cell numbers across samples. For all evaluated parameters, a strong positive correlation with cellular load was observed (R^2^ ≈ 0.91–0.99). The highest concordance was detected for protein concentration measured in the post-extraction precipitate (R^2^ = 0.99), while slightly lower R^2^ values were observed for summed XIC (R^2^ ≈ 0.91–0.98). Pairwise correlations between all replicate-averaged quantitative parameters exceeded 0.99, indicating exceptionally high internal consistency across measurements.

**Figure 3:**
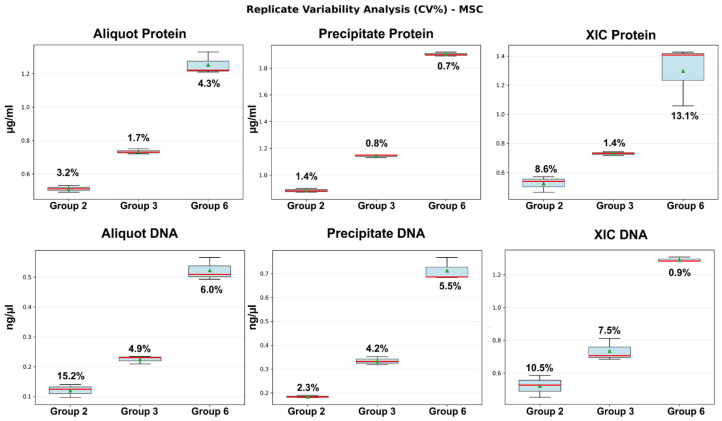
Box-and-whisker plots showing median (red line), mean (green triangle), and coefficients of variation (CV, %) annotations for six quantitative metrics across three cell loading levels (2, 3, and 6 million cells per sample) in MSC samples. Metrics include protein and DNA concentrations measured in aliquots of cell suspension and precipitates, as well as XIC Protein (protein precipitate extraction) and XIC DNA (DNA precipitate extraction).

Reproducibility within each cellular load group (2, 3, and 6 million cells per sample) was assessed from technical replicates (Figure 3). For the majority of parameters, variability was low, with coefficients of variation ranging from 0.7% to 6.0% for protein (whether measured from suspension aliquots or precipitates) and precipitate-derived DNA, and from 0.9% to 13.1% for XIC, depending on the cell loading level.

Subsequently, we evaluated correlations between chromatographic peak areas and the corresponding normalization parameters. Table 3 summarizes the mean R2 values calculated across all detected metabolites, together with the percentage distribution of metabolites falling within each correlation ranges. In terms of mean R^2^, all normalization methods demonstrated satisfactory performance (>0.80), reflecting a strong overall association between metabolite responses and the corresponding normalization factors. However, examining the distribution of R^2^ values by metabolite class reveals that protein-based approaches tend to yield somewhat lower correlations than DNA-based normalization, with metabolites more frequently falling in the 0.75–0.90 range rather than the >0.95 group characteristic of DNA. Interestingly, a similar pattern was observed for cell count normalization, which may point to inaccuracies introduced during sample pooling at the preparation stage. For protein-based normalization specifically, the reduced correlations may reflect greater susceptibility to technical variability, despite the higher robustness to biological variability demonstrated in the previous experiment. XIC-based normalization, in contrast, consistently yielded high correlations under controlled conditions, which is expected given that this metric effectively functions as an internal normalization factor.

**Figure 4:**
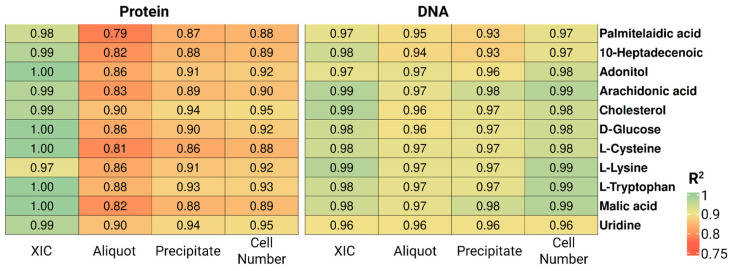
Coefficients of determination (R^2^) between peak areas of 11 selected metabolites and six normalization parameters—protein and DNA concentrations in precipitate and aliquots of cell suspension, plus total extracted ion current and number of cells from protein-extract and DNA-extract for MSC samples.

Figure 4 shows R^2^ values for a representative set of ten metabolites spanning distinct chemical classes, evaluated across six normalization factors. DNA quantified directly from cell suspension aliquots—without prior precipitation—yielded the highest correlations, with R^2^ values of 0.95–0.99 for the majority of metabolites, indicating a near-linear relationship between DNA concentration and metabolite signal intensity. Comparable performance was observed for protein quantified from the aliquot (R^2^ = 0.8–0.9). Across all biological normalization markers, performance was markedly improved relative to the first experiment.

To assess whether the observed correlation patterns were influenced by any specific metabolite class, R^2^ values were further stratified by chemical class (amino acids, peptides and analogs; carbohydrates and carbohydrate conjugates; fatty acids and conjugates; Supplementary Table S1). Class-averaged R^2^ values for the most populated groups deviated from the global means by less than ~5%, indicating that the trends reported in Table 3 reflect a consistent behavior across chemically diverse metabolites rather than a class-specific effect.

The coefficients of determination for the 11 representative metabolites spanning different chemical classes (Figure 4) broadly confirm the overall trends reported in Table 3. Protein-based approaches showed markedly lower correlations than DNA-based normalization, whereas XIC-based normalization consistently maintained strong correlation patterns across all metabolite classes examined.

Normalization based on summed XIC yielded exceptionally high correlations, with R^2^ values of 0.97–0.99 for most metabolites. However, these results should be interpreted with caution in the context of pooled starting material. Unlike biomass-derived metrics (DNA or protein content), which reflect genuine differences in biological material between aliquots, XIC represents an integral property of the entire chromatographic profile. In this experimental setting, the high XIC correlations primarily reflect the quality and homogeneity of the aliquoting process rather than the independent suitability of these parameters for normalization factor under conditions of uncontrolled biological variability.

Protein and DNA quantified from the post-extraction precipitate also demonstrated high R^2^ values (0.95–0.99 for DNA and 0.75–0.95 for protein). The modest reduction in performance relative to aliquot-based measurements likely reflects partial protein and DNA losses incurred during precipitation and centrifugation steps.

To complement the correlation analysis, coefficients of variation were calculated for the 11 selected metabolites after applying each normalization strategy (Table 4). Across these compounds, normalization generally reduced between-sample dispersion, with normalized peak areas clustering more tightly around their respective means. Normalization to biomass-derived parameters shows a clear advantage of DNA-based metrics over protein-based ones in this dataset. This is broadly consistent with the findings from a previous study [9], in which DNA was identified as the preferred normalization target. Normalization to DNA in the precipitate yielded the lowest dispersion of normalized peak areas (mean CV ≈ 17%), closely followed by DNA in the aliquot of cell suspension (≈19%), whereas normalization to protein in the aliquot and in the precipitate results in noticeably higher mean CVs (≈25% and ≈28%, respectively). For 9 of the 11 metabolites, one of the two DNA-based measures (aliquot of cell suspension or precipitate) provided the lowest CV among all biological normalization options, with precipitate DNA marginally outperforming aliquot DNA. Protein-based normalization was optimal only for a limited subset of compounds, notably hydrophobic metabolites such as cholesterol and 9-octadecenoic acid, and protein from the precipitate did not emerge as the best variant for any metabolite in this particular comparison. XIC-based normalization also produced comparatively low CVs across many metabolites, which is consistent with the design of this experiment: all samples originated from the same pooled biological material and were processed using identical extraction and analytical workflows. Under such controlled conditions, XIC functions as an effective technical scaling factor that efficiently equalizes signal intensities, even though, as discussed above, its utility as a primary normalization metric is more limited in biologically heterogeneous settings. Cell count normalization yielded the lowest mean CV among the 11 selected metabolites; however, like XIC-based normalization, its universal applicability remains a matter of debate.

The substantial improvement in correlation strength—from moderate values in the first experiment (R^2^ ≈ 0.50–0.70) to near-linear relationships in the second experiment (R^2^ ≈ 0.80–0.90)—indicates that the dominant source of variability in the initial experiment was intrinsic biological heterogeneity of the HepG2 samples. This heterogeneity is likely aroused from the use of reagents from different manufacturers during cell culture, compounded by unavoidable physiological variation accumulated during extended passaging.

It is important to note that, in the context of normalization protocol development, this experiment represents an intermediate validation step. The primary objective of normalization is to compensate for variability arising from differences in the amount of biological material grown under realistic, partially uncontrolled conditions. In the present experiment, such variability was intentionally eliminated through pooling. Nevertheless, these results provide critical insight into the technical capabilities and analytical limits of the normalization approach. Specifically, they demonstrate that in the absence of biological variability, quantitative performance can approach R^2^ ≈ 1.00, confirming that the methodology itself imposes no fundamental analytical constraints. Conversely, the presence of biological variability does not preclude effective normalization in the studied system. As demonstrated below, a subset of metabolites consistently recurring across experiments exhibits strong correlations between signal intensity and normalization factors. These metabolites often have clear biological origins and may serve as reference markers for assessing normalization quality.

### 2.4. Robustness of Normalization Strategies Under Controlled Biological Variability in HepG2 Metabolomics

Having demonstrated that protein content, DNA content, and total extracted ion current can serve as effective normalization factors under conditions of limited or fully suppressed biological variability, we next examined how these metrics perform when modest, experimentally controlled heterogeneity is introduced between independent cultures—conditions that more closely reflect realistic in vitro settings. To this end, three parallel HepG2 cultures were grown under identical conditions in separate dishes, and samples spanning a broad range of cell amounts were collected from each culture. This design simultaneously expanded the number of data points for assessing analytical linearity and allowed for an explicit evaluation of whether the proposed biomass metrics can compensate for between-culture differences in metabolic state and growth history.

From each of the three cell suspensions, a series of five samples was generated by aliquoting, yielding approximate cell numbers of 1, 2, 3, 4, and 5 million cells per replicate. This experimental design served two complementary objectives: to increase the resolution of the biomass–signal relationship by providing more data points than in previous experiments, and to assess the extent to which inter-culture variability could be mitigated by the proposed normalization strategies.

**Figure 5:**
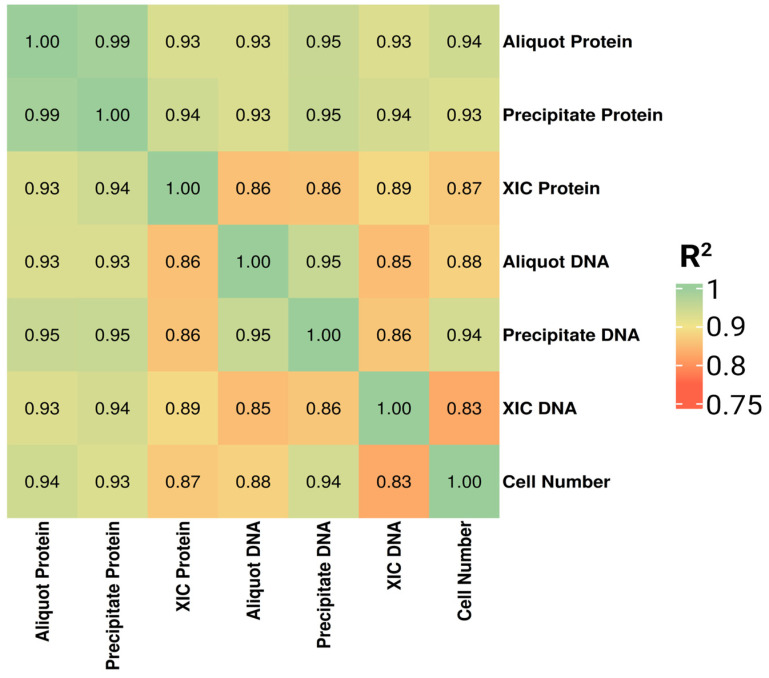
Heatmap of correlation coefficients between six methods for determining the amount of biological material (XIC, total protein, and DNA determined from cell precipitate and cell suspension aliquots).

Overall, all approaches for estimating biological material content retained strong correlations between the normalization factor and the calculated cell number (R^2^ ≥ 0.98, Figure 5). High concordance was preserved among all normalization metrics, as reflected by strong coefficients of determination between the respective parameters. Nevertheless, overall agreement was modestly reduced relative to the previous experiment, likely reflecting the introduction of controlled biological variability across independent cultures.

**Figure 6:**
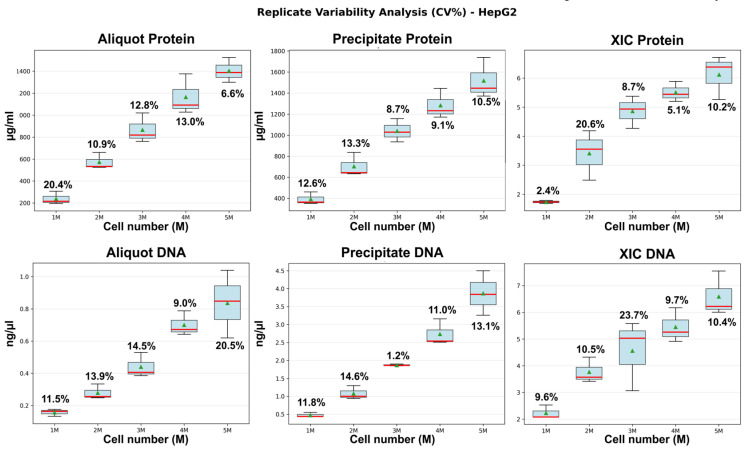
Box-and-whisker plots showing median (red line), mean (green triangle), and CV% annotations for six quantitative metrics across five cell loading levels (1, 2, 3, 4 and 5 million cells per sample) in HepG2 cell line samples. Metrics include protein and DNA concentrations in aliquots of cell suspension and precipitates, as well as total extracted ion chromatogram peak areas: XIC Protein (protein precipitate extraction) and XIC DNA (DNA precipitate extraction).

As shown in Figure 6, coefficients of variation were markedly lower when protein and DNA levels were quantified from the post-extraction cellular precipitate, remaining within 15% for both protein and DNA. XIC, in contrast, exhibited the highest variability, exceeding 20% at certain biomass levels—likely because this metric captures not only differences in biological material quantity but also sample-to-sample variation in metabolomic composition introduced by the deliberate biological heterogeneity in this experimental design.

These findings further support the superior robustness of precipitate-based normalization over XIC under conditions of biological variability. 

Across the full metabolite set, the same leading trends were maintained: precipitate-based normalization yielded high correlations for a greater fraction of metabolites compared with the alternative strategies.

**Figure 7:**
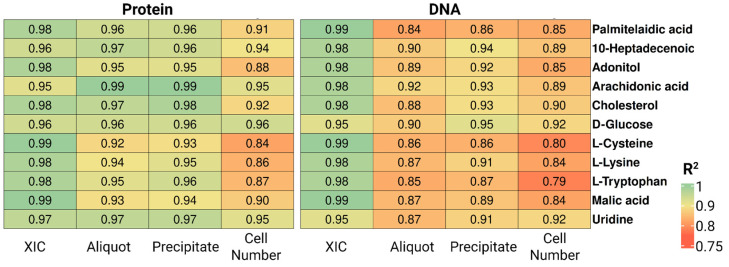
Coefficients of determination (R^2^) between peak areas of 11 selected metabolites and six normalization parameters—protein and DNA concentrations in precipitate and aliquots of cell suspension, plus total extracted ion current and number of cells from protein-extract and DNA-extract for HepG2 samples.

From a correlation perspective, all protein-based normalization approaches in this experiment yielded strong associations between the normalization factor and metabolite signal intensity, with R^2^ values of 0.90–0.99 for the majority of metabolites (Table 5, Figure 7). At the same time, DNA-based normalization showed a marked decline in correlation performance relative to the experiment conducted without biological variability, suggesting greater susceptibility to confounding effects and raising questions about its reliability in real biological samples. Notably, DNA-based normalization yielded higher average coefficients of variation for normalized metabolite signals than protein-based approaches (26% vs. 20%). For both protein and DNA, aliquot-based quantification produced lower CVs than precipitate-based quantification (protein: 19.5% vs. 21.2%; DNA: 22.7% vs. 29.8%), a pattern that held consistently across the selected metabolites (Table 6). Protein-based methods additionally maintained superior overall reproducibility. In the experiment incorporating biological variability, cell count-based normalization consistently underperformed relative to protein-based approaches, yielding both higher CVs and lower R^2^ values. We attribute this to inaccuracies in cell quantification, which are likely amplified under less controlled experimental conditions.

Variability was predominantly driven by samples containing low amounts of biological material. Specifically, for samples comprising 3–5 million cells normalized to total precipitate protein, the mean CV was 8%, which is 13 percentage points lower than that calculated across all five loading groups. A similar trend was observed across all other normalization strategies. Together, these findings indicate that normalization performance deteriorates at low cell concentrations in suspension, regardless of the method applied.

When controlled but non-zero biological variability was introduced across independently cultured HepG2 populations, all biomass-derived metrics—protein, DNA, XIC, and cell count—remained strongly linear with nominal cell number, confirming the stability of the analytical workflow over a broad dynamic range. Quantitative performance nevertheless differed between normalization strategies: biopolymer-based metrics derived from the post-extraction pellet yielded lower CVs and a greater proportion of metabolites with R^2^ > 0.9, whereas XIC and cell count proved more sensitive to metabolic heterogeneity and counting inaccuracies. Among individual approaches, aliquot-based protein and DNA measurements generally minimized dispersion, while pellet-derived protein offered a favorable balance between robustness at higher biomass loads and resilience to biological variability. All methods showed reduced performance at low cell numbers. Taken together, under realistic conditions with moderate biological heterogeneity, protein-based normalization—whether from aliquots or pellets—represents the most reproducible and broadly applicable scaling basis for GC × GC-MS metabolomic data from adherent HepG2 cells, while DNA- and cell count-based metrics demand stricter control of assay precision and counting accuracy.

## 3. Materials and Methods

### 3.1. Cell Lines

The human hepatoblastoma cell line (HepG2) was purchased from Merck, Darmstadt, Germany. Primary mesenchymal stem cells (MSCs) were obtained from in-house cell collection of IBMC and used at passages 3–5.

After thawing, the cells were cultured under standard conditions (37 °C, 5% CO_2_, 100% humidity) in complete growth medium consisting of DMEM/F12 (PanEco, Moscow, Russia) supplemented with 10% FBS (Gibco, Waltham, MA, USA; Dia-M, Moscow, Russia; HyClone, Parramatta, Australia) and 100 U/mL penicillin/streptomycin (PanEco, Russia).

Cells were expanded in 75 cm^2^ flasks to 80% confluency. For passaging, the cells were detached by incubating with 0.25% trypsin–EDTA solution (PanEco, Moscow, Russia) for 5–10 min at 37 °C, washed twice with Hank’s balanced salt solution (PanEco, Moscow, Russia), and subcultured at a 1:3 ratio in the growth medium.

### 3.2. Preparing the Sample for Protein and DNA Analysis from the Aliquot

Cells were detached as described above, washed twice with Hank’s balanced salt solution (PanEco, Moscow, Russia), resuspended in PBS (Gibco, Waltham, MA, USA), and counted in a standard cell counter (Goryaev chamber, Minimed, Bryansk, Russia) according to the manufacturer’s instructions.

A series of 100 μL cell suspension samples containing 1, 2, 3, 4, 5, and 6 million cells were prepared. Depending on the experiment, different subsets of these concentrations (e.g., 2, 3, and 6 million or 1–5 million cells) were subjected to immediate cell lysis. Lysis was performed by three freeze–thaw cycles using liquid nitrogen, followed by ultrasonic treatment (80 kHz, 5 min, room temperature, Elmasonic P, Singen, Germany).

From each 100 μL lysate, a 20 μL aliquot was collected and subsequently used for protein and DNA quantification. Briefly, the 20 μL aliquot was diluted fivefold with deionized water, after which 20 μL of the resulting solution was withdrawn for downstream protein analysis. The remaining volume was reserved for DNA extraction.

The collected aliquot was diluted 1:1 with an 0.1 M NaOH and used for protein analysis. Protein concentration was determined according to the manufacturer’s instructions using the Pierce BCA Protein Assay Kit (Thermo Scientific, Waltham, MA, USA).

Genomic DNA was isolated from the remaining 80 μL of the cell lysate. DNA extraction and purification were performed according to the manufacturer’s protocol using the ExtractDNA Blood & Cells kit (Evrogen, Moscow, Russia). Prior to analysis, purified DNA was diluted tenfold with deionized water, and 10 μL of the resulting solution was used for DNA quantification. Quantitative analysis was performed fluorometrically using the QuDye dsDNA HS assay kit (Lumiprobe, Moscow, Russia) on a Qubit 4 fluorometer (Thermo Fisher Scientific, Waltham, MA, USA).

### 3.3. Sample Preparation for Protein and DNA Analysis from the Precipitate After Extraction

To control the amount of biological material entering downstream analyses, two 35 μL aliquots were collected from the remaining cell suspension and subjected to metabolite extraction. Extraction was performed using one of three ice-cold solvent mixtures: acetonitrile/isopropanol/water (3:3:2, *v*:*v*:*v*), methanol/chloroform/water (7:2:1, *v*:*v*:*v*), or methanol/water (8:2, *v*:*v*).

To each aliquot of lysate, 1 mL of extraction solvent was added, followed by incubation for 5 min at 4 °C and centrifugation (14,000× *g*, 2 min). The supernatant was collected in two portions of 450 μL each and dried completely in a vacuum centrifuge at 30 °C (Concentrator plus, Eppendorf, Hamburg, Germany). One portion was used for metabolomic analysis, while the second was retained as a backup.

The post-extraction precipitate from one of the two replicates was dried to completeness in a vacuum centrifuge at 30 °C and subsequently reconstituted in 200 μL of 0.1 M NaOH. A 20 μL aliquot was used for the determination of total protein content by BCA assay as described above.

The post-extraction precipitate from the second replicate was similarly dried and subjected to DNA isolation. The purified DNA was diluted 20-fold with deionized water, and a 10 μL aliquot was used for DNA quantification. Quantitative analysis was performed fluorometrically using the QuDye dsDNA HS assay kit (Lumiprobe, Moscow, Russia) on a Qubit 4 fluorometer (Thermo Fisher Scientific, Waltham, MA, USA).

### 3.4. Quantitative Analysis of Protein and DNA

Total protein was quantified using the Pierce™ BCA Protein Assay Kit (Thermo Fisher Scientific, Waltham, MA, USA) according to the manufacturer’s protocol, with absorbance measured at 562 nm on EzDrop 1000 spectrophotometer (Blue-Ray Biotech, New Taipei City, Taiwan). Both lysate aliquots and post-extraction precipitates were analyzed.

DNA was isolated using the ExtractDNA Blood & Cells kit (Evrogen, Moscow, Russia) following the manufacturer’s protocol. DNA was quantified using the QuDye dsDNA HS assay kit (Lumiprobe, Moscow, Russia) on a Qubit 4 fluorometer (Thermo Fisher Scientific, Waltham, MA, USA).

### 3.5. Preparing the Sample for Metabolomic Analysis

The samples prepared according to the protocol described in Section 2.3 were then treated with 10 μL of freshly prepared methoxyamine hydrochloride (20 mg/mL in pyridine) and incubated at 30 °C for 90 min in ThermoMixer C (Eppendorf, Hamburg, Germany) at 1300 rpm. Subsequently, derivatization was performed by adding 91 μL of N-methyl-N-(trimethylsilyl)trifluoroacetamide (MSTFA) with a mixture of fatty acid methyl esters (FAME) and incubating the samples at 37 °C for 30 min in a thermomixer at 1300 rpm.

Derivatized samples were analyzed immediately by two-dimensional gas chromatography–mass spectrometry (GC × GC–MS). Analytical reproducibility was monitored throughout acquisition by tracking the retention times and peak areas of the FAME standards included in each sample preparation batch.

### 3.6. GC × GC-MS Analysis

GC × GC–MS analyses were performed using a 7890B gas chromatographic system (Agilent Technologies, Santa Clara, CA, USA) coupled to a Pegasus BT 4D time-of-flight mass spectrometer (LECO Corporation, St Joseph, MI, USA) and equipped with an L-PAL3 autosampler (CTC Analytics AG, Zwingen, Switzerland).

Samples (1 μL) were injected via a glass inlet liner (Restek, Bellefonte, PA, USA) in split mode (10:1), with helium (grade 6.0) as the carrier gas at a constant flow rate of 1 mL/min. The oven temperature was initially set to 60 °C with an equilibration time of 1 min, followed by a temperature ramp of 10 °C/min to a final temperature of 280 °C, which was held for 12 min. Separation was achieved on a 30 m Rxi-5Sil MS primary column coupled to a 3 m Rxi-17Sil MS secondary column (both Restek, Bellefonte, PA, USA).

The MS transfer line temperature was set to 280 °C, with acquisition delay of 350 s. The ion source temperature was maintained at 250 °C. Mass spectra were acquired over a mass range of *m*/*z* 35–700 with electron ionization at 70 eV. The acquisition rate was set to 200 spectra per second.

### 3.7. Metabolome Data Processing

GC × GC–MS data were processed using ChromaTOF software (v. 5.58; LECO Corporation, St Joseph, MI, USA). Compound identification was performed by matching mass spectra against the NIST and LECO–Fiehn Rtx5 libraries, using a similarity threshold of 700. Retention indices were calculated using the FAME mix. For each feature, the peak area was averaged across the three technical replicates, after which metabolites—identified as their underivatized parent molecules—were assigned InChIKey structural identifiers. Features corresponding to the same metabolite were aggregated by summing their peak areas prior to downstream analysis.

A metabolite was retained if it met the following criterion: peak area for this metabolite was at least 4-fold above the blank in at least two out of three experimental replicates with the highest cell number per sample, in at least one of the two extraction sets (prepared for protein or DNA determination in the precipitate). For XIC-based normalization, the total extracted ion current was calculated per GC × GC-MS replicate as the cumulative signal of all selected and reliably identified metabolites, then averaged within each biological sample.

Statistical analysis and visualization were performed using Python 3.11.9 with the following libraries: ‘pandas’ (v2.2.2) and ‘numpy’ (v1.26.4) for data processing, ‘scipy.stats’ (v1.13.1) for linear regression and correlations, and ‘matplotlib.pyplot’ and ‘seaborn’ for plotting.

## 4. Conclusions

In this study, we performed a comprehensive comparison of normalization strategies for GC × GC–MS metabolomic data obtained from MSC and HepG2 cell lines. The most widely used approaches were evaluated, including quantification of total protein and double-stranded DNA from both aliquots and post-extraction precipitates, as well as normalization based on extracted ion current and number of cells, in combination with three widely used extraction solvent systems. Under controlled technical variability, metabolite signal–biomass relationships were predominantly strong, while the moderate correlations observed under realistic cell culture conditions were shown to arise primarily from biological rather than technical variability. The methanol/chloroform/water (7:2:1) extraction system provided an optimal balance between metabolome coverage and robustness of biomass-dependent signals, with protein and DNA quantified from the cellular precipitate exhibiting comparatively low coefficients of variation and high reproducibility.

Based on these analyses, we conclude that normalization to total protein quantified from the post-extraction cellular precipitate, obtained using the methanol/chloroform/water (7:2:1) system, represents the most appropriate normalization approach for metabolomic profiling of cell lines. This approach consistently outperformed or matched alternative normalization schemes, while remaining experimentally straightforward and providing an intuitive readout of the amount of biological material analyzed. XIC-based normalization also performed well across conditions, and its key practical advantage is that it does not require any additional experimental measurements beyond the metabolomic acquisition itself. In this context, XIC-based normalization is best suited as a sensitive indicator of aliquoting quality and analytical stability, and as a pragmatic fallback option when biomass measurements are not feasible, rather than as a primary normalization factor under conditions of pronounced biological variability. Normalization to cell count likewise yielded high correlation levels under highly controlled (“idealized”) conditions, but this metric deteriorated noticeably in samples that contained appreciable biological variability. The metabolomic workflow proposed here has the potential to substantially improve data quality and to facilitate more robust inter-laboratory comparisons in cellular metabolomics.

## Figures and Tables

**Table 1:** Average coefficients of determination (R^2^) between chromatographic peak areas and normalization factors (protein and DNA concentrations in precipitates and aliquots of cell suspension, summed XIC for selected metabolites and number of cells), along with the number of metabolites exhibiting R^2^ > 0.75.

Type of Analyte	Solvent	Aliquot of Cell Suspension		Precipitate		XIC		Number of Cells	
		Mean R^2^	Metabolites with R^2^ > 0.75	Mean R^2^	Metabolites with R^2^ > 0.75	Mean R^2^	Metabolites with R^2^ > 0.75	Mean R^2^	Metabolites with R^2^ > 0.75
Protein	methanol/chloroform/water (7:2:1)	0.51	32	0.66	51	0.71	60	0.70	57
	methanol/water (8:2)	0.51	29	0.47	30	0.56	18	0.52	18
	acetonitrile/isopropanol/water (3:3:2)	0.46	15	0.48	28	0.57	15	0.45	19
DNA	methanol/chloroform/water (7:2:1)	0.25	1	0.64	48	0.64	31	0.67	49
	methanol/water (8:2)	0.42	20	0.62	50	0.73	61	0.68	52
	acetonitrile/isopropanol/water (3:3:2)	0.38	0	0.54	31	0.68	50	0.60	48

**Table 2:** Coefficients of variation (CV,%) between samples, average concentrations of protein and DNA.

Solvent	Protein Determination (BCA)		DNA Determination (PicoGreen)	
	CV Between Samples, %	Average Concentration, μg/mL	CV Between Samples, %	Average Concentration, ng/μL
methanol/chloroform/water (7:2:1)	0.99	956.54	4.35	2.07
methanol/water (8:2)	0.12	865.66	3.68	2.05
acetonitrile/isopropanol/water (3:3:2)	0.25	720.99	5.58	1.87

**Table 3:** Average R^2^ values between metabolite signals and the corresponding normalization factors, along with the distribution of R^2^ values across six correlation levels (expressed as a percentage of the total number of metabolites, n = 90).

	Protein in Aliquot	Protein in Precipitate	XIC from Protein Sample	Number of Cells for Protein Sample	DNA in Aliquot	DNA in Precipitate	XIC from DNA Sample	Number of Cells for DNA Sample
mean R^2^	0.82	0.87	0.97	0.88	0.92	0.93	0.95	0.94
R^2^ > 0.95	0%	1.1%	91.2%	1.1%	67%	69.2%	85.7%	80.2%
0.9 < R^2^ < 0.95	4.4%	37.4%	6.6%	49.5%	18.7%	18.7%	4.4%	9.9%
0.75 < R^2^ < 0.9	87.9%	59.3%	0%	47.3%	8.8%	6.6%	5.5%	4.4%
0.5 < R^2^ < 0.75	6.6%	1.1%	1.1%	1.1%	4.4%	4.4%	3.3%	4.4%
R^2^ < 0.5	1.1%	1.1%	1.1%	1.1%	1.1%	1.1%	1.1%	1.1%

**Table 4:** Coefficients of variation (CV, %) of normalized peak areas for 11 selected metabolites after applying six normalization approaches: protein and DNA quantified in aliquots of cell suspension and precipitates, extracted ion current (XIC) and number of cells from the protein-oriented and DNA-oriented extracts for MSC samples.

Metabolite	Protein in Aliquot, CV After Normalization	Protein in Precipitate, CV After Normalization	XIC from Protein Sample, CV After Normalization	Number of Cells from Protein Sample, CV After Normalization	DNA in Aliquot, CV After Normalization	DNA in Precipitate, CV After Normalization	XIC from DNA Sample, CV After Normalization	Number of Cells from DNA Sample, CV After Normalization
Palmitelaidic acid	38.5	41.0	30.5	27.3	24.1	25.4	39.7	31.8
10-Heptadecenoic acid	19.5	21.4	11.5	11.8	35.6	21.6	12.8	14.1
Adonitol	33.6	36.5	27.4	23.6	22.1	27.1	36.9	31.8
Arachidonic acid	35.1	37.9	28.4	24.8	14.8	13.3	27.7	20.1
Cholesterol	9.7	11.9	3.7	10.2	23.5	16.7	8.7	7.7
D-Glucose	22.6	25.5	16.0	15.1	13.8	14.4	20.7	16.1
L-Cysteine	20.2	22.7	11.7	13.8	13.9	14.1	17.9	13.2
L-Lysine	28.4	31.1	24.1	21.5	18.9	12.6	11.8	7.6
L-Tryptophan	19.9	22.9	13.9	11.6	14.8	12.4	15.8	10.6
Malic acid	24.5	27.1	16.5	15.7	10.1	12.6	22.0	15.6
Uridine	23.6	27.0	19.5	14.9	15.1	16.3	18.8	15.7

**Table 5:** Average R^2^ values between metabolite signals and the corresponding normalization factors, along with the distribution of R^2^ values across six correlation levels (expressed as a percentage of the total number of metabolites, n = 141.

	Protein in Aliquot	Protein in Precipitate	XIC from Protein Sample	Number of Cells for Protein Sample	DNA in Aliquot	DNA in Precipitate	XIC from DNA Sample	Number of Cells for DNA Sample
mean R^2^	0.89	0.89	0.91	0.83	0.81	0.84	0.92	0.77
R^2^ > 0.95	23.4%	25.5%	47.5%	2.1%	0.0%	4.2%	49.6%	0.7%
0.9 < R^2^ < 0.95	41.1%	39.7%	25.5%	22.7%	9.9%	21.8%	27.7%	3.5%
0.75 < R^2^ < 0.9	29.8%	29.8%	19.1%	62.4%	78.2%	62.0%	17.0%	60.3%
0.5 < R^2^ < 0.75	3.5%	2.8%	5.6%	9.9%	9.2%	9.9%	4.3%	33.3%
R^2^ < 0.5	2.1%	2.1%	2.1%	2.8%	2.8%	2.1%	1.4%	2.1%

**Table 6:** Coefficients of variation (CV, %) of normalized peak areas for 11 selected metabolites after applying six normalization approaches: protein and DNA quantified in aliquots of cell suspension and precipitates, extracted ion current (XIC) and number of cells from the protein-oriented and DNA-oriented extracts for HepG2 samples.

Metabolite	Protein in Aliquot, CV After Normalization	Protein in Precipitate, CV After Normalization	XIC from Protein Sample, CV After Normalization	Number of Cells from Protein Sample, CV After Normalization	DNA in Aliquot, CV After Normalization	DNA in Precipitate, CV After Normalization	XIC from DNA Sample, CV After Normalization	Number of Cells from DNA Sample, CV After Normalization
Palmitelaidic acid	18.8	10.4	7.8	15.2	26.6	33.4	8.6	20.3
10-Heptadecenoic acid	12.9	13.5	14.6	13.8	21.7	24.5	15.9	15.7
Adonitol	18.6	26.1	26.8	25.5	22.9	20.1	23.7	20.7
Arachidonic acid	12.2	12.8	16.8	12.1	18.8	22.3	18.8	15.3
Cholesterol	11.7	12.4	13.8	14.1	22.3	22.9	17.8	15.5
D-Glucose	12.9	13.9	14.9	12.0	22.7	29.3	14.3	14.8
L-Cysteine	16.3	17.8	13.9	21.0	26.2	30.5	12.2	21.4
L-Lysine	31.8	13.9	10.6	22.9	30.9	43.3	5.9	27.0
L-Tryptophan	13.9	21.5	22.4	22.9	27.1	24.2	25.5	25.3
Malic acid	17.9	15.2	11.2	16.4	25.7	33.3	8.7	20.2
Uridine	19.5	8.5	8.7	13.2	24.7	29.6	14.2	16.1

## Data Availability

The data supporting the reported results, including GC × GC-MS metabolomic peak areas for all detected features, measured concentrations of total protein and DNA (from both cell suspension aliquots and extraction precipitates), full correlation matrices (R^2^ values) across all normalization factors and metabolites, chemical class annotations for detected compounds, and associated metadata, are available in Supplementary Table S1.

## Supplementary Materials

The following supporting information can be downloaded at: https://www.mdpi.com/article/10.3390/ijms27073219/s1.

## Abbreviations

The following abbreviations are used in this manuscript:

XIC	Extracted ion current
CV	Coefficients of variation
MSCs	Mesenchymal stem cells
GC	Gas chromatography
MS	Mass spectrometry
